# Supplementary material for: Altered Cerebral Processing of Videos in Children with Motor Dysfunction Suggests Broad Embodiment of Perceptual Cognitive Functions
Source: J Pers Med. 2022 Nov 4;12(11):1841. doi: 10.3390/jpm12111841 (PMC9697218; doi:10.3390/jpm12111841)
Supplement: Supplementary file 1 [file jpm-12-01841-s001.zip › jpm-1959749-supplementary.pdf]

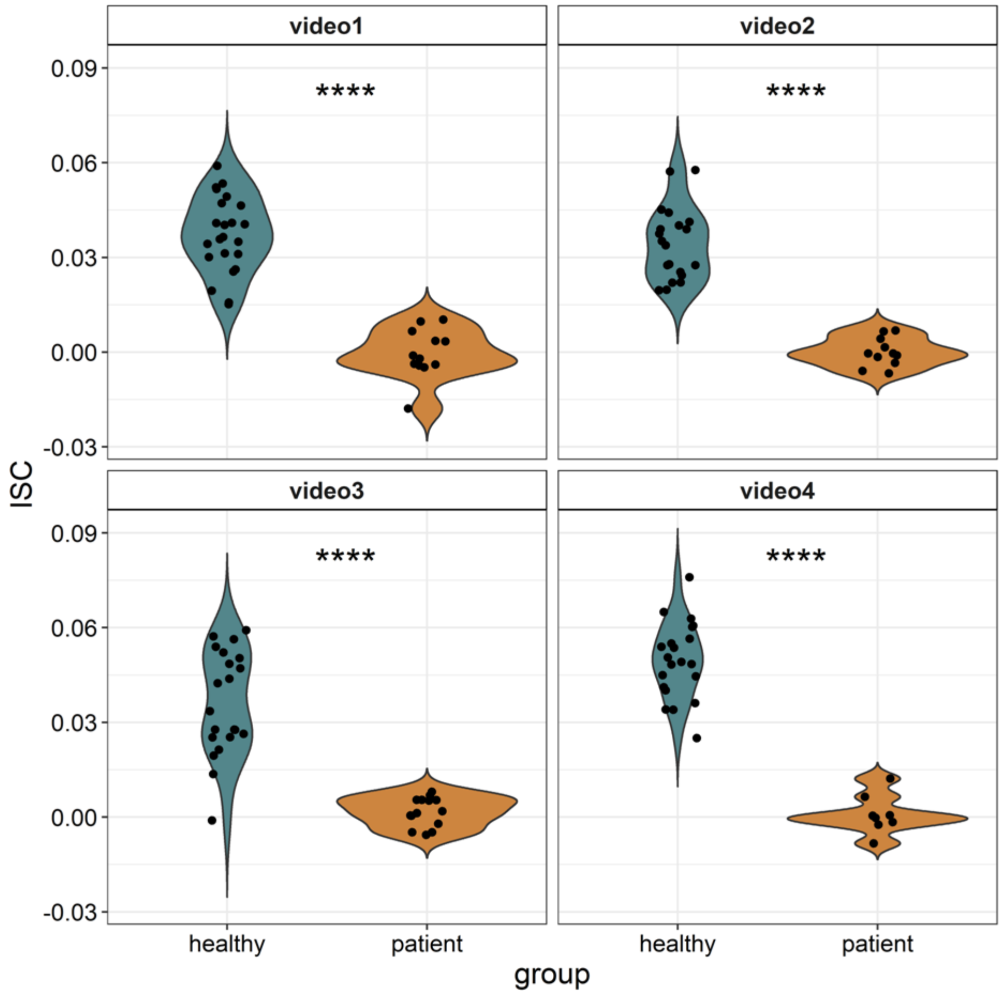

Supplementary Figure S1. *Leave-one-out ISC as the sum of the three strongest components for each video separately. Each subject corresponds to a dot representing how similar this subject's brain activity was to the group of healthy children. Pairwise differences were assessed with T-tests after confirming that the observations are normally distributed, with Shapiro-Wilk test. *p < .05, **p < .01, ***p < .001, ****p < .0001, after Bonferroni adjustment.*

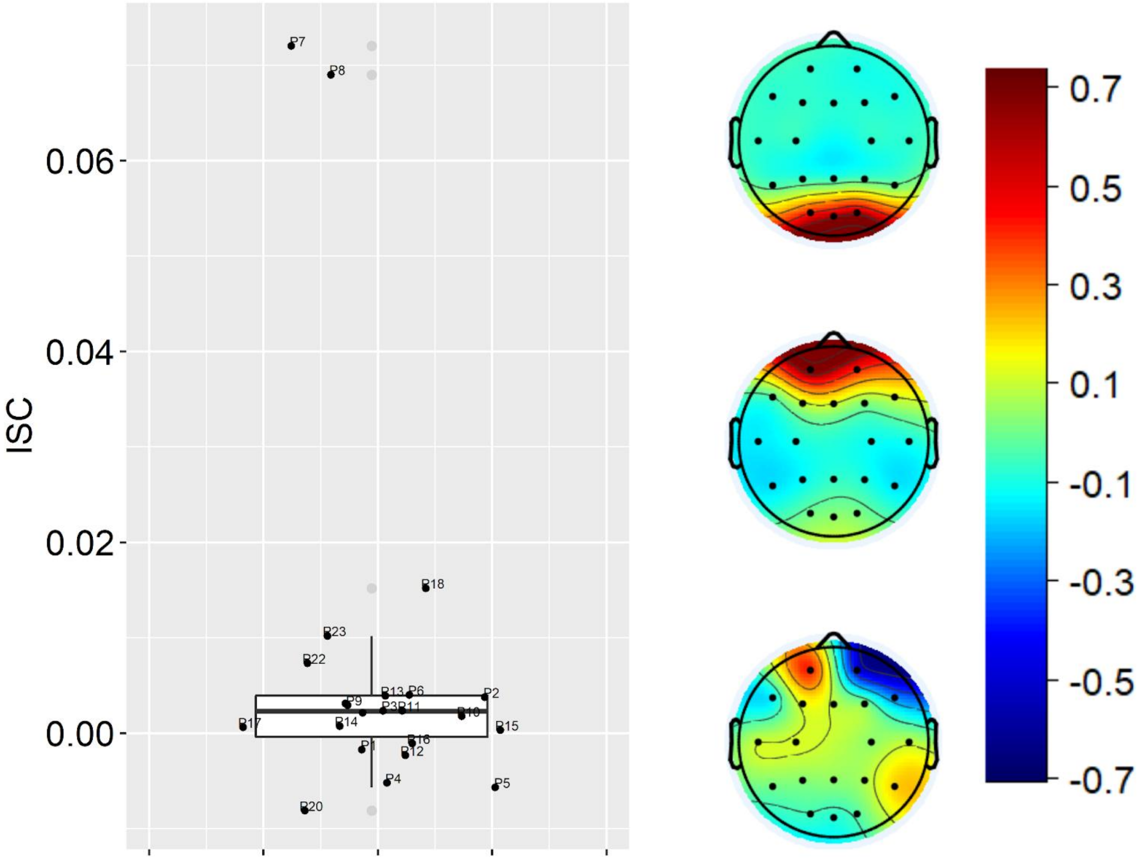

Supplementary Figure S2. *Within-AMC patients ISC. Each subject corresponds to a dot representing how similar this subject's brain activity was to all other patients, on average across videos. A One-Sample Wilcoxon Signed Rank Test revealed that the ISC is significantly higher than zero (p=.018, effect size=0.438). We also tested the hypothesis that ISC is greater than 0 after excluding patients P7 and P8 as possible outliers. The effect dropped from significan to marginal (p=.056, effect size=0.353).*

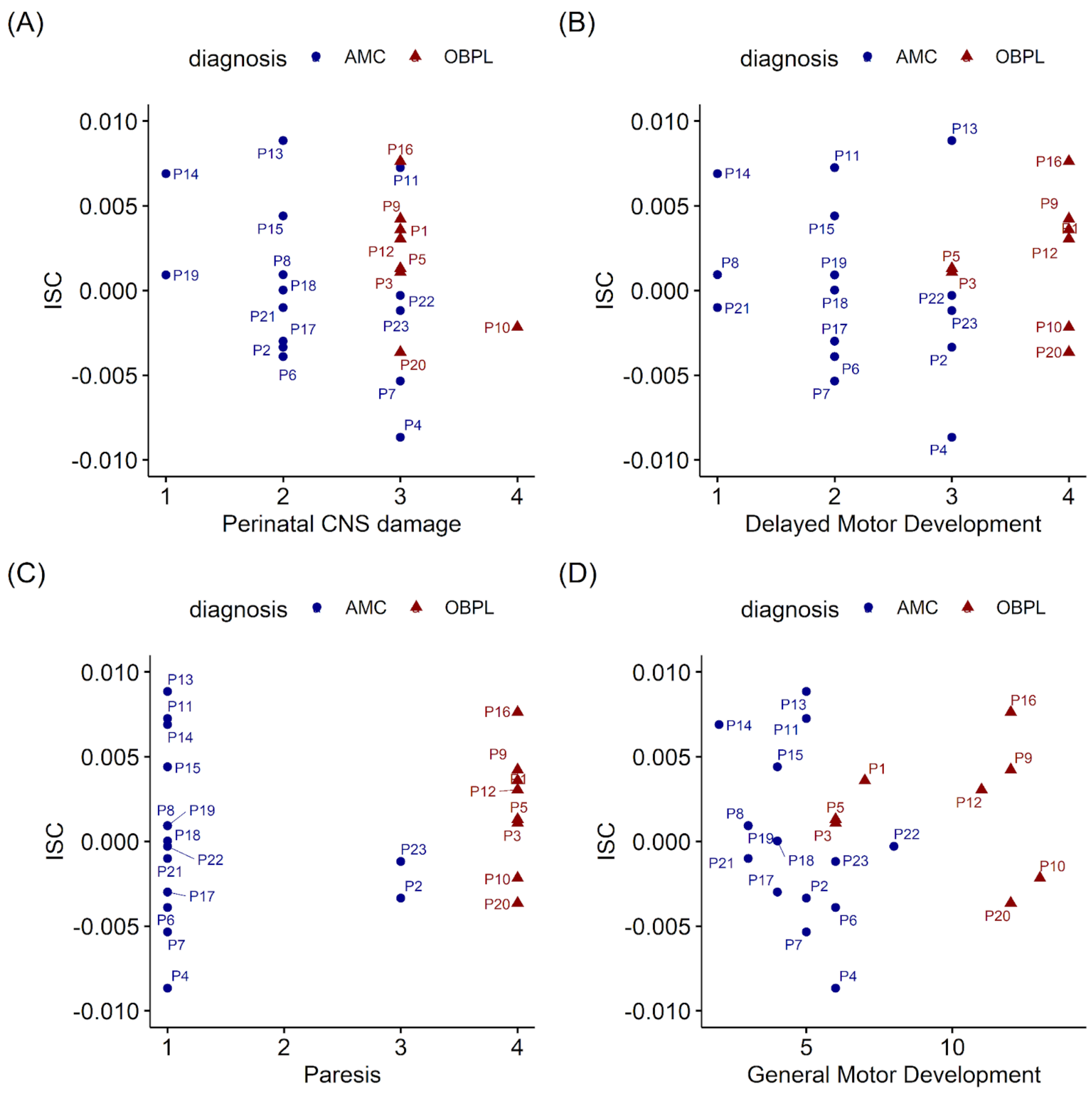

Supplementary Figure S3. *Association between diagnostic measurements of patients and patients' ISC to healthy controls. In all panels, ISC is presented as the sum of the three strongest components, averaged across videos. Each patient's ID is given on the plot for reference with Supplementary Table S1. ISC was not associated with the diagnostic scores of AMC patients (τ(ISC,CNS)=-.312, p=.149; τ(ISC,DMD)=-.220, p=.305; τ(ISC,Paresis)=-.230, p=.308; r(ISC,GMD)=-.349, p=.202) nor with that of OBPL patients (τ(ISC,CNS)=-.357, p=.275; τ(ISC,DMD)=.218, p=.505; r(ISC,GMD)=-.073, p=.864; τ(ISC,Paresis)=NA since all OBPL patients scored 4 in Paresis).*

Effect	Est.	S.E.	t val.	d.f.	p
(Intercept)	-0.001	0.011	-0.138	4.914	0.896
Gander Male	-0.008	0.003	-2.825	8.042	0.022
Age	-0.001	0.001	-1.111	7.734	0.3
Movement Arm	0.042	0.124	0.335	13.732	0.743
Movement Leg	0.014	0.18	0.077	41.386	0.939
Movement Both	-0.001	0.096	-0.008	6.589	0.994
Diagnosis OBPL	0.01	0.009	1.02	4.925	0.355
Attention span	0.003	0.002	1.069	4.292	0.341
Auditory memory	0	0.002	0.06	5.333	0.954
Visual memory	0	0.001	-0.378	3.926	0.725
Formation of generalization	0	0.002	0.021	4.01	0.985
DMD	-0.001	0.003	-0.216	4.177	0.839
CNS damage	-0.004	0.003	-1.174	3.998	0.306
GMD	0.001	0.001	1.116	5.027	0.315
Paresis	-0.003	0.003	-0.833	4.252	0.449
Movement Arm : Diagnosis OBPL	-0.032	0.107	-0.299	13.728	0.77
Movement Leg : Diagnosis OBPL	-0.004	0.156	-0.023	41.349	0.982
Movement Both : Diagnosis OBPL	0.013	0.083	0.163	6.586	0.876
Movement Arm : Attention span	-0.001	0.03	-0.04	13.721	0.969
Movement Leg : Attention span	-0.002	0.043	-0.055	41.347	0.957
Movement Both : Attention span	-0.008	0.023	-0.345	6.559	0.741
Movement Arm : Auditory memory	0.001	0.018	0.03	13.72	0.976
Movement Leg : Auditory memory	0.003	0.026	0.112	41.357	0.911
Movement Both : Auditory memory	0.004	0.014	0.278	6.563	0.79
Movement Arm : Visual memory	0.001	0.012	0.084	13.71	0.934
Movement Leg : Visual memory	-0.001	0.018	-0.075	41.319	0.941
Movement Both : Visual memory	0	0.009	0.036	6.556	0.972
Movement Arm : Formation of generalization	-0.001	0.022	-0.06	13.717	0.953
Movement Leg : Formation of generalization	0.002	0.032	0.054	41.331	0.957
Movement Both : Formation of generalization	-0.001	0.017	-0.049	6.562	0.962
Movement Arm : DMD	-0.011	0.042	-0.267	13.734	0.793
Movement Leg : DMD	-0.003	0.061	-0.054	41.382	0.957
Movement Both : DMD	0.002	0.032	0.067	6.563	0.948
Movement Arm : CNS damage	0	0.043	0.01	13.706	0.992
Movement Leg : CNS damage	-0.007	0.063	-0.104	41.329	0.917
Movement Both : CNS damage	0.005	0.034	0.158	6.551	0.879
Movement Arm : GMD	0.001	0.015	0.056	13.719	0.956
Movement Leg : GMD	0.001	0.021	0.066	41.36	0.948
Movement Both : GMD	-0.004	0.011	-0.322	6.553	0.758
Movement Arm : Paresis	0.012	0.039	0.316	13.731	0.757
Movement Leg : Paresis	0.001	0.057	0.015	41.348	0.988
Movement Both : Paresis	-0.003	0.03	-0.104	6.588	0.92

Supplementary Table S1. Fixed effects from mixed effects linear models of each variable's effect on patients' ISC with the healthy cohort.

Score	Paresis	CNS damage	DMD
5	no limb disturbance	-	-
4	one limb disturbance	no CNS damage	normal development
3	two limbs disturbance	CNS damage, but with no severe motor and speech disorders	The patient can either walk independently or has managed to walk independently later than normal, they can sit down independently
2	three limbs disturbance	CNS damage with motor and speech disorders, which are expected to evolve in the future	The patient cannot walk independently but it is possible for them to walk with special devices. The patient can stand and walk with support, they can sit down independently
1	four limbs disturbance	CNS damage with severe motor and speech disorders	The patient cannot walk independently, they can stand with support but they cannot move with support, they can sit down either independently or with support

Supplementary Table S2. *Description of the clinical assessment scores.*

	Group	
	Healthy	Patient
Video 1	23	12
Video 2	20	11
Video 3	21	14
Video 4	22	8

Supplementary Table S3. *Number of subjects and patients who watched each video stimulus.*

	Movement			
	arm	leg	both	neither
Video 1	17%	14%	23%	46%
Video 2	27%	18%	12%	43%
Video 3	20%	18%	17%	45%
Video 4	13%	33%	21%	33%

Supplementary Table S4. *Proportion of arm movement, leg movement, simultaneous arm and leg movement (both) and neither arm nor leg movement (neither), shown in each video. Notably, these four sets of recording samples are disjoint.*
